# On Piles, Their Diagnosis and Treatment*Read before the Harveian Society.

**Published:** 1876-04

**Authors:** Alfred Cooper


### ON PILES, THEIR DIAGNOSIS AND TREATMENT.*

By ALFRED COOPER, F.R.C.S..

Piles is the popular name for the second most common disease of the rectum; not the most common, as one would think, by every disease of that region being called piles. It is common, on asking a patient who comes before me for the first time, at a certain special hospital to which I am attached, what he complains of, to receive as answer, "Piles." "Who told you so?" "My

*Read before the Harveian Society.

doctor." "Did he make any examination of the part?" "No." "What did he give you?" "A brown ointment to use and some brown stuff to take." Gentlemen, I have seen fistula, fissure, mucous tubercles, stricture, cancer, foreign bodies, (such as fish-bones) and pruritus, treated by these two universal remedies—the ointment causing, as you may imagine, generally an increase to the pain from which the patient was already suffering enough. The rectum is almost, if not quite, the most sensitive part of the body, and its diseases cause as much suffering as any other part, and therefore we cannot be too careful in our diagnosis.

To diagnose "piles" is very easy, you would say, but I have seen several cases where mistakes have been made by surgeons attached to our large hospitals, simply from a want of care in their diagnosis. One case, I remember, which I asked my colleague, Mr. Allingham, to see with me, in which a celebrated hospital surgeon was going to operate for internal piles on a patient who had cancer of the rectum in a very advanced condition. Also another case which occurred lately, in which I was asked to remove some external piles, said to be so by a surgeon of one of our large hopitals, who had made no internal examination, and ordered lead lotion externally. I found a large polypus attached to the front wall of the rectum, but no external piles. To diagnose an external pile is a very easy matter; if you just separate the buttocks and look you will find, if there be one, a round dark-looking swelling, having a smooth surface, and feeling very tense in its consistence, the patient complaining of great pain when the swelling is touched. An external pile is venous in its character, and consists, as a rule, of a clot of coagulated venous blood held in a small vein, or extravasated into its neighboring tissue, and is generally caused by some stoppage to the return of blood through the liver, and is commonly produced by errors in diet, and sitting in damp places or on wet saddles.

It is common to call all pieces of redundant skin in this part external piles, which is quite a mistake, as they more frequently indicate mischief inside the bowel.

*To Diagnose Internal Piles.*—If a patient tells you that, on going to the closet, something always protrudes, you had better give him an enema of warm water, and ask him to go to the closet, and when the water has come away to put a towel to the part and come and lie down on the sofa. Then you can see what it is, and, as a rule, you will find one or more tumors, which your patient will say came down when at the closet, which may bleed or not, and which go up of their own accord after keeping quiet a short time; this is the first stage of internal piles. In the second stage they come down, and have always to be replaced. In the third stage they come down on the least exertion, and it is difficult to keep them up when replaced. In this stage, not unfrequently, they come down and remain down, and nature herself operates on them by strangulating them with her ligature, the sphincter, and they slough away.

Internal piles are composed of large dilated blood vessels, united by connective tissue and covered with mucous membrane, which usually bleeds at the slightest touch; they are sometimes more arterial, sometimes more venous in their character. They are caused like the external by some stoppage in the circulation of the blood, and are produced also, like them, by error of diet and a sedentary life; but, unlike the external piles, they are often hereditary.

Gentlemen, I hope I have shown how very easy it is to diagnose "piles," and I would most strongly suggest that you always make a proper visit, and tactile examination of the rectum before giving an opinion, and not attempt to diagnose diseases of this part by looking at your patient's tongue, as was the custom of a certain American doctor, who once told me he could usually tell when a patient had rectal disease, or any other, by certain marks on his tongue.

We now come, gentlemen, to the treatment of piles.

We must first try and remove, if possible, the causes, by attending to any errors in diet, or mode of living, and regulating the action of the bowels; the liver, too, should receive our special attention. Our next treatment should be local, cold applications, as a rule, being better than warm, except for external piles, in pregnancy, when warm applications are best. For external piles, I prefer astringent lotions to ointments, and never use gall ointment, as I have seldom seen it do any good when ordered by other surgeons, and it is a very dirty application. Ointments are more useful when any abrasion of the skin or ulceration co-exists with an external pile. Should this treatment fail to give relief, I incise the pile and let out the clot, and then use cold applications, and I have never seen any ill consequences follow the treatment.

The treatment for internal piles must also be to try and remove anything causing obstruction to the return of blood by paying particular attention to the regular action of the bowels, by getting them to act before going to rest (a great comfort to patients who have to work during the day), to bathe the piles when they come down, with cold or iced water, and always carefully to return them with a little simple ointment. By these means patients may go on for a long time, without sufficient inconvenience to cause an operation to become necessary.

We now come to the question, When ought we to operate to remove internal piles? *Never*, if the patient is a full-blooded person, who has suffered from congestion of the head, which is always relieved by the bleeding from the piles, or in patients who have periodical hemorrhage from their piles, with relief to themselves. I have seen two deaths follow soon after the operation where this rule had not been adhered to; the patients both died from apoplexy. Be careful to examine with the finger to find that no more serious disease exists. The cases for operative interference are those in which the patient has become quite emaciated from loss of blood; also, those cases where they cause great discomfort by the constant irritating discharge from them, and nearly always in the third stage, when they remain down, and prevent a patient moving about.

There are three ways of destroying internal piles; by the ligature, by the clamp and actual cautery, and by nitric acid. I have seen the ligature used by my colleagues, and have used it myself now for eleven years, without a bad case, nor have we had a case of pyæmia (in over four thousand cases at St. Mark's

Hospital) occurring after the operation. The operation we do at St. Mark's Hospital, and which was handed down to us by Mr. Salmon, is to pull down with a fork, or vulsellum, the tumor, and with a pair of scissors divide it from the skin, cutting in the groove you will always find between the skin and mucous membrane, and in a straight line with the bowel. Then your assistant lifts up the pile, and you place your ligature in the cut you have made; your assistant then pulls the pile down over the ligature, and you tie it at its base and return it, or cut it off as you like, and remove any external redundant skin you may think necessary, being careful not to remove too much. The ligatures generally come away in four or five days, and the patient is about again in seven or ten days. The old method of transfixing the pile with double ligatures and tieing each half is still practiced, I believe, by some of our hospital surgeons; but it is not, I think, so good an operation. The clamp I seldom use, as I have not found any of the advantages claimed for it over the ligature. Nitric acid I have left off using, except in slight cases (when it astringes them up very successfully), as I have had two cases of severe hemorrhage following its use when the sloughs came away; and my colleagues have also had severe hemorrhage occurring after the application of nitric acid.

Gentlemen, if this paper will cause any of my professional brethren to be a little more careful in their examination of the rectum, which still seems to be a "terra incognita" in the domain of surgery, it will more than repay the trouble I have taken in putting these few words together.—*London Med. Ex.*

